# Inducible *Slc7a7* Knockout Mouse Model Recapitulates Lysinuric Protein Intolerance Disease

**DOI:** 10.3390/ijms20215294

**Published:** 2019-10-24

**Authors:** Susanna Bodoy, Fernando Sotillo, Meritxell Espino-Guarch, Maria Pia Sperandeo, Aida Ormazabal, Antonio Zorzano, Gianfranco Sebastio, Rafael Artuch, Manuel Palacín

**Affiliations:** 1 Institute for Research in Biomedicine (IRB Barcelona), The Barcelona Institute of Science and Technology (BIST), 08028 Barcelona, Spain; fsotilro@gmail.com (F.S.); antonio.zorzano@irbbarcelona.org (A.Z.); 2 Department of Biosciences, University of Vic, 08500 Vic, Spain; 3 Centro de Investigación Biomédica en Red de Enfermedades Raras (CIBERER), 08003 Barcelona, Spain; aormazabal@sjdhospitalbarcelona.org (A.O.); Rartuch@sjdhospitalbarcelona.org (R.A.); 4 Sidra Medicine, Translational Medicine Department, Doha 26999, Qatar; mespinoguarch@sidra.org; 5 Department of Translational Medicine, Section of Pediatrics, Federico II University of Naples, 80138 Naples, Italy; mariapiasperandeo@yahoo.it (M.P.S.); gianfrancosebastio@gmail.com (G.S.); 6 Department of Clinical Biochemistry, Hospital Sant Joan de Déu (HSJD), 08950 Esplugues del Llobregat, Spain; 7 Institut de Recerca Sant Joan de Déu, 08950 Esplugues de Llobregat, Spain; 8 Centro de Investigación Biomédica en Obesidad (CIBEROB), 28029 Madrid, Spain; 9 Department of Biochemistry and Molecular Biomedicine, Faculty of Biology, University of Barcelona, 08028 Barcelona, Spain

**Keywords:** LPI, rare disease, amino acid transporter, y^+^LAT1, hypoargininemia, hyperammonemia, pulmonary alveolar proteinosis

## Abstract

Lysinuric protein intolerance (LPI) is a rare autosomal disease caused by defective cationic amino acid (CAA) transport due to mutations in *SLC7A7*, which encodes for the y^+^LAT1 transporter. LPI patients suffer from a wide variety of symptoms, which range from failure to thrive, hyperammonemia, and nephropathy to pulmonar alveolar proteinosis (PAP), a potentially life-threatening complication. Hyperammonemia is currently prevented by citrulline supplementation. However, the full impact of this treatment is not completely understood. In contrast, there is no defined therapy for the multiple reported complications of LPI, including PAP, for which bronchoalveolar lavages do not prevent progression of the disease. The lack of a viable LPI model prompted us to generate a tamoxifen-inducible *Slc7a7* knockout mouse (*Slc7a7^−/−^*). The *Slc7a7^−/−^* model resembles the human LPI phenotype, including malabsorption and impaired reabsorption of CAA, hypoargininemia and hyperammonemia. Interestingly, the *Slc7a7^−/−^* mice also develops PAP and neurological impairment. We observed that citrulline treatment improves the metabolic derangement and survival. On the basis of our findings, the *Slc7a7^−/−^* model emerges as a promising tool to further study the complexity of LPI, including its immune-like complications, and to design evidence-based therapies to halt its progression.

## 1. Introduction

Lysinuric protein intolerance (LPI, MIM222700) is a rare autosomal recessive disorder caused by defective cationic amino acid (CAA) transport due to mutations in *SLC7A7*, which encodes for the y^+^LAT1 protein [1,2]. y^+^LAT1 heterodimerizes with CD98hc (also named 4F2hc), and mediates the exchange of CAA with neutral amino acids plus sodium, mainly at the basolateral membrane of epithelial cells of the kidney and intestine [3]. The y^+^LAT1-CD98 heterodimer also mediates arginine transport in non-polarized cells, such as macrophages and monocytes, and is essential for the correct function of these cells (e.g., proper inflammatory response) [4]. However, the implications of the depletion of y^+^LAT1 in macrophages and monocytes require further attention.

Over 200 LPI patients have been reported worldwide, with isolated populations in Finland and Japan showing the highest prevalence (1:60,000 and 1:57,000, respectively) [5,6,7]. In addition, clusters of patients have been reported in southern Italy [8], North Africa [9] and Turkey [10,11] and sporadic cases in Greece, Pakistan [12], China [13] and Spain [1]. Although up to 50 mutations have been described [9], all the Finnish patients share the same point mutation, c.895-2A > T, which generates an acceptor splice site error that leads to a frameshift deletion and the loss of y^+^LAT1 activity [1,2].

LPI patients suffer impaired absorption and reabsorption of CAA in the intestine and kidney, respectively, manifested by hyperexcretion of CAA, which results in low CAA levels in plasma. Typically, plasma concentrations of lysine, arginine and ornithine in patients are one-third to one-half the normal means values, but occasionally may be within the normal range [14]. Shortage of arginine and ornithine, providers of the carbon skeleton of the urea cycle, causes episodes of hyperammonemia [15]. Patients remain asymptomatic while breast-feeding and symptoms like vomiting, diarrhea, seizures and coma appear after weaning, when high-protein food intake starts [14]. LPI symptoms range from hypotonia, bone fractures, hepatosplenomegaly, mental retardation, hyperlipidemia, end-stage renal disease to immune-like disorders (e.g., hemophagocytic lymphohistiocytosis, pulmonary alveolar proteinosis (PAP) and glomerulonephritis) [16,17]. Of these complications, PAP is the most lethal condition and has caused the death of several LPI patients in recent years [18]. No genotype–phenotype has been demonstrated, as patients with the same mutation (e.g., Finnish mutation) show a wide range of symptoms [17].

First-line treatment of LPI consists of a protein restriction diet and mostly oral supplementation of L-citrulline (2.5–8.5 g/day), a neutral amino acid and intermediary metabolite of the urea cycle that can be converted to arginine and ornithine, and whose bioavailability is only marginally compromised by defective y^+^LAT1 [14]. In some instances, ammonia scavengers are necessary when episodes of hyperammonemia appear [17]. Due to the complexity of the disease, treated LPI patients also suffer from a wide range of immune and hematological complications [19,20,21]. There are no effective treatments for these alterations and the potential negative impact of citrulline is under debate [16]. 

PAP is characterized by alveolar spaces filled with lipoproteinaceous material because the surfactant clearance by alveolar macrophages (AMs) is impaired [22]. AMs appear enlarged, foamy and filled with lipids [18], and about two thirds of patients have interstitial alterations in chest radiographs [14]. LPI-associated PAP has no defined treatment because bronchoalveolar lavages are palliative solutions but do not prevent disease progression. The pathogenesis of this condition remains unclear although some studies point to alterations of bone marrow-derived monocytes, which differentiate to mature AMs and are responsible for catabolizing the surfactant [23,24]. 

LPI patients also suffer from neurological impairment mainly due to hyperammonemia episodes after protein-rich meals. Moreover, cases with acute encephalopathy, seizures and coma, persistent hypotonia or developmental disability have also been recorded [19,25]. Hyperammonemia causes astrocyte swelling and brain edema [26,27], and *Slc7a7* is also reported to be required by macrophages that give rise to microglia in the brain [28]. Unfortunately, the underlying mechanisms of the pathophysiology of LPI complications are unknown.

The *null Slc7a7* mice are perinatally lethal with intrauterine growth restriction [29]. In this study, only 16 *Slc7a7 null* homozygote mice were born out of >600 expected newborns, and only two survived on a low protein diet and citrulline supplementation but finally died when exposed to a normal diet.

Given that there is an urgent need for a viable LPI model to study the physiopathology of the disease and design evidence-based therapies, here we present a conditional inducible mouse model that recapitulates the main features of human LPI disease.

## 2. Results

### 2.1. The Slc7a7^−/−^ Mouse Model 

**Figure 1:**
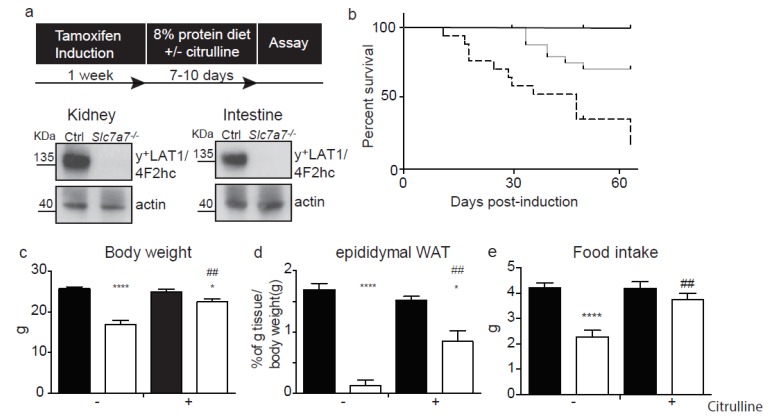
LPI mouse model and citrulline treatment. (**a**) Schematic representation of timeframes for tamoxifen induction and citrulline treatment. Twelve-week-old animals were fed a tamoxifen diet for 1 week. The diet was then changed to 8% protein with or without citrulline supplementation. Western blot is of total membranes from kidney and intestine against y^+^LAT1-CD98 heterodimer (135 kDa) from *Slc7a7^loxP/loxP^ Cre^−^* (*Slc7a7^−/−^*) and *Slc7a7^loxP/loxP^ Cre*^+^ mice. β-actin was used as a loading control; (**b**) Survival plot at 60 days with 13 animals per group; *Slc7a7^loxP/loxP^* animals fed an 8% protein diet with citrulline supplementation (grey line) showed enhanced survival vs. *Slc7a7^−/−^* animals without citrulline supplementation (dashed line). Black line represents control animals (*Slc7a7 ^loxP/loxP^* without Cre expression treated with tamoxifen diet and 8% protein diet); (**c**–**e**) Body, epydidymal white adipose tissue weight and food intake of tamoxifen induced *Slc7a7^loxP/loxP^ Cre*^−^ (white bars) compared with control *Slc7a7^loxP/loxP^ Cre*^+^ (black bars) animals with or without citrulline supplementation as indicated on the X axis. Data correspond to the mean ± SEM of 9 animals. Statistical significance * *p* < 0.05, **** *p* < 0.0001 vs. control. ## *p* < 0.01 vs. citrulline treatment was analysed using a Student’s *t*-test.

Gene targeting was used to insert loxP and FRT sites flanking exons 3 and 4 of murine *Slc7a7* gene and a Neo cassette, respectively. *Slc7a7^loxP/+^* and *FLP^+/−^* mice were bred to eliminate the neomycin selection cassette (Supplementary Figure S1a). Chimeras were generated in the C57BL/6-129 background and more than 12 backcrossed alternating sexes were used to purify the mixed colony to a pure C57BL6 generation. Mice with the floxed allele were then paired with the human ubiquitin C promoter-Cre (UBC-Cre) mice [30] to generate *Slc7a7^lox/+;^ Cre^+/−^* progeny. Specific PCR amplification confirmed *Slc7a7^+/+^*, *Slc7a7^loxP/+^* and *Slc7a7^loxP/loxP^* with Cre expression (Supplementary Figure S1b). With this approach, Cre would mediate the excision of floxed exons 3 and 4, compromising translation from the ATG starting codon to part of the fourth transmembrane domain (Supplementary Figure S1c), a nuclear part of the folding of the protein [31,32,33]. Mice submitted to the tamoxifen diet for one week showed ablation of the y^+^LAT1 protein in kidney and intestine (Figure 1a), the two main tissues that express the transporter [2].

*Slc7a7^−/−^* mice in a low protein diet showed a reduced survival (~50%; 1-month after tamoxifen induction) compared to control mice (Figure 1b). *Slc7a7^−/−^* mice had dramatic decreases in body and white adipose tissue (WAT) weights after 7–10 days of tamoxifen induction (Figure 1c–d). In contrast, no differences were observed in liver, skeletal muscle or kidney weights (Supplementary Figure S2a–c). In addition, *Slc7a7^−/−^* mice showed a lower food intake as well as less feces depositions (Figure 1e and Supplementary Figure S2d). Citrulline supplementation in drinking water (1 mg/mL) improved body and WAT weights and food intake parameters, thereby increasing survival to 100% 1-month after tamoxifen induction (Figure 1b–e). These data confirm that *Slc7a7^−/−^*, as LPI patients, required citrulline supplementation to meliorate the phenotype [14]. 

### 2.2. Renal and Intestinal Phenotype

**Figure 2:**
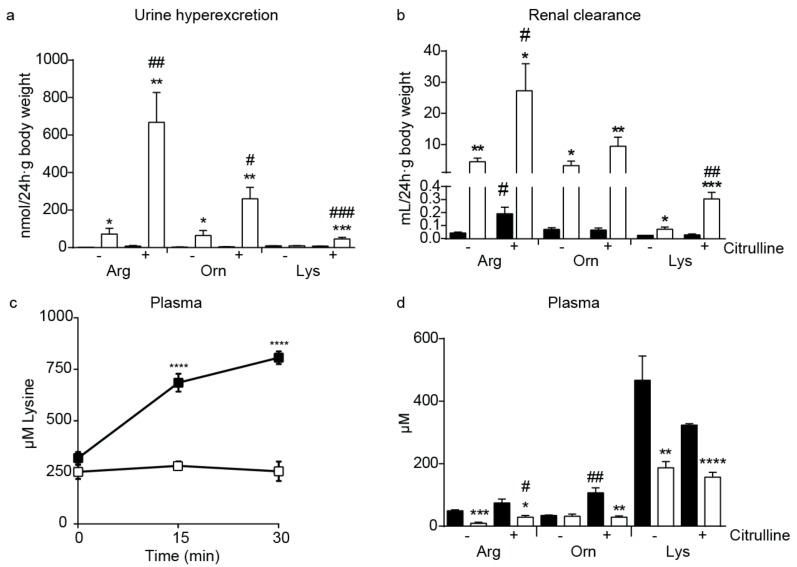
*Slc7a7^−/−^* recapitules all the main hallmarks of LPI. (**a**) Hyperexcretion of cationic amino acids in *Slc7a7^−/−^*. Cationic amino acid (CAA) concentration in urine relative to creatinine concentration at 24 h and body weight of 12-month-old animals; (**b**) Renal clearance of cationic amino acids calculated from Supplementary Equation S1; (**c**) Malabsorption of lysine; (**d**) CAA concentration in plasma. Control (black bars or squares) and *Slc7a7^−/−^* (white bars or squares) animals were analyzed. Data corresponds to the mean ± SEM of 6 animals per group. Statistical significance * *p* < 0.05, ** *p* < 0.01, *** *p* < 0.001, **** *p* < 0.0001 vs. control. # *p* < 0.05, ## *p* < 0.01 vs citrulline treatment was analyzed using a Student’s *t*-test.

The *Slc7a7^−/−^* mice showed urine hyperexcretion, increased renal clearance and reduced tubular reabsorption of CAA (Table 1 and Figure 2a,b), indicating a primary defect of renal reabsorption of CAA in the tubule. Urine excretion of arginine and ornithine were higher than that of lysine (Figure 2a) with the reabsorption defect in kidney tubules being more severe for arginine and ornithine than for lysine in *Slc7a7^−/−^* mice. 

Estimation of the tubular reabsorption of amino acids was based on urine excretion, plasma levels of amino acids and the glomerular filtration rate (GFR, Supplementary Equation S2), determined by the creatinine urine excretion and plasma levels (Supplementary Equation S3). GFR and creatinine urine excretion was reduced in *Slc7a7^−/−^* mice, but with normal creatinine plasma levels 7–10 days after tamoxifen-induced ablation of y^+^LAT1 (Supplementary Figure S3). Hematoxylin/eosin staining showed no glomerular histological alterations (data not shown) at this time point. Citrulline treatment decreased GFR in control, but not in *Slc7a7^−/−^* mice. This effect is attributed to dilation of efferent vessels by NO produced from newly synthetized arginine from citrulline in the tubular epithelial cells [34]. In all, these data show altered glomerular function, and suggest a tendency of the LPI mouse to renal insufficiency. Indeed, glomerulonephritis and end-stage renal disease are late complications in human LPI [14]. Additional studies at longer periods of time after y^+^LAT1 ablation will be necessary to assess whether *Slc7a7^−/−^* mice develop renal insufficiency. 

In order to test CAA malabsorption, mice were submitted to an oral gavage of lysine. The blood concentration of lysine tripled 30 min after the oral gavage in control mice while it remained steady in the LPI model (Figure 2c). In contrast, glucose absorption was not affected in *Slc7a7^−/−^* mice (Supplementary Figure S4). Therefore, the lack of a y^+^LAT1 transporter causes a specific malabsorption of lysine, most probably extended to all CAA in mice.

Defects in both intestinal absorption and renal reabsorption of CAAs would decrease their plasma levels. Indeed, *Slc7a7^−/−^* mice showed diminished concentrations of arginine and lysine, but unaltered ornithine plasma concentrations (Figure 2d). Interestingly, citrulline treatment improved hypoargininemia in *Slc7a7^−/−^* mice (Figure 2d). Animals treated with citrulline showed increased urine hyperexcretion and renal clearance of CAA (Figure 2a,b) along with negative tubular reabsorption for arginine and ornithine in *Slc7a7^−/−^* mice (Table 1). This observation is explained by absorption of citrulline in the intestine and conversion to arginine and ornithine via renal enzymes (argininosuccinate synthetase and argininosuccinate lyase) upon reaching the epithelial proximal tubule cells [35], increasing the plasma concentration of these CAA. Part of the de novo synthetized arginine and ornithine is excreted in urine, contributing to the negative values of tubular reabsorption. 

LPI patients commonly show increased plasma concentrations of neutral amino acids (AA^0^) such as glutamine, alanine, serine, citrulline, methionine and proline [14]. Similarly, *Slc7a7^−/−^* mice presented increased AA^0^ plasma levels (Supplementary Table S1). *Slc7a7^−/−^* mice also presented moderate hyperexcretion, incremented renal clearance and diminished tubular reabsorption for some AA^0^ (Supplementary Table S2–4). Thus, the incremented AA^0^ plasma levels are most probably due to metabolic alterations in LPI (e.g., compromised uptake of AA^0^ by y^+^LAT1/CD98hc in cells expressing this transporter). 

### 2.3. Hyperammonemia and Neurological Symptoms

**Figure 3:**
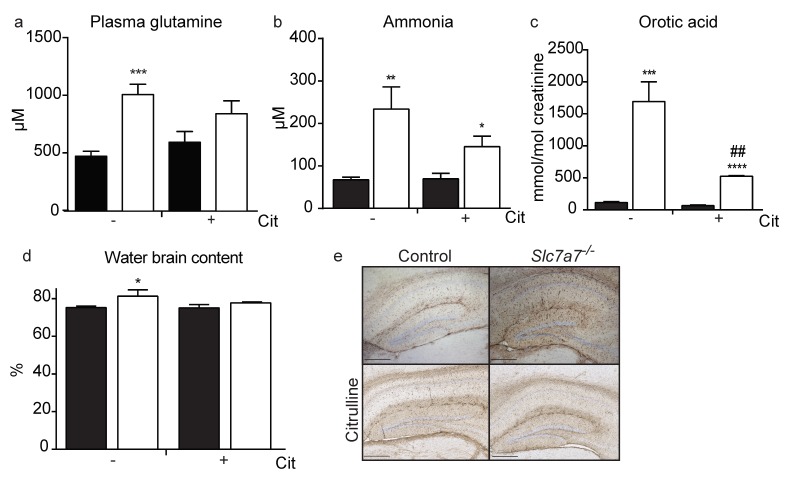
*Slc7a7^−/−^* mice show hyperammonemia and brain alterations. (**a**) *Slc7a7^−/−^* mice (white bars) have higher glutamine concentration in plasma; (**b**) hyperammonemia and (**c**) higher levels of orotic acid in urine compared to controls (black bars); (**d**) brain water content is also increased in *Slc7a7^−/−^* mice. Citrulline treatment (+ Cit) improved all the parameters measured. Data corresponds to the mean ± SEM of 6 mice per group (a to d). Statistical significance * *p* < 0.05, ** *p* < 0.01, *** *p* < 0.001, **** *p* < 0.0001 vs. control. ## *p* < 0.01 vs. citrulline treatment was analyzed using a Student’s *t*-test. (**e**) Representative images of glial fibrillary acidic protein (GFAP) immunochemistry of hippocampus sections from control and *Slc7a7*^−/−^ mice treated (2 control and 4 *Slc7a7*^−/−^ mice, lower panels) or not (9 control and 4 *Slc7a7*^−/−^ mice) with citrulline. Scale bar = 400 µm

Recurrent hyperammonemic encephalopathy occurs in LPI patients [19]. Thus, patients present a range of behavioral symptoms such as drowsiness (limited speech, poor eye contact, restless) and coma [25]. It is suggested that the main cause of these neurological signs is a defect in the urea cycle caused by the low availability of arginine and ornithine in the hepatocytes [17]. *Slc7a7^−/−^* mice showed increased concentrations of ammonium and the ammonium-sink glutamine in plasma, as well as orotic acid in urine (Figure 3a,b), supporting a urea cycle defect in the LPI mouse. Such defects, which are caused by a shortage of intermediates, as it is the case of LPI and hyperornithinemia–hyperammonemia–homocitrullinuria (HHH) syndrome due to defective mitochondrial ornithine translocase ORC, accumulate carbamyl phosphate, which is converted into orotic acid for urine excretion [36]. The hyperexcretion of orotic acid in the LPI mouse suggests that compromised availability of intermediates is at the base of the urea cycle defect. Indeed, as in LPI patients [16], supplementation with citrulline, an intermediate of the cycle, partially recovered glutamine concentration in plasma, hyperammonemia and urine excretion of orotic acid (Figure 3c). 

Hyperammonia and an excess of glutamine impairs astrocyte function and morphology by increasing osmotic pressure and finally causing brain edema [37]. *Slc7a7^−/−^* animals showed increased water content in the brain and astrocytosis (increased GFAP staining) (Figure 3d,e). Interestingly, citrulline supplementation normalized brain edema and recovered astrocytosis (Figure 3d,e). Thus, citrulline supplementation restored argininemia, the urea cycle defect (ammonemia, glutaminemia and orotic aciduria) and brain edema. These data support the notion that shortage of urea cycle intermediates underlie the neurological complications of LPI in *Slc7a7^−/−^* mice. 

### 2.4. Lung Involvement

**Figure 4:**
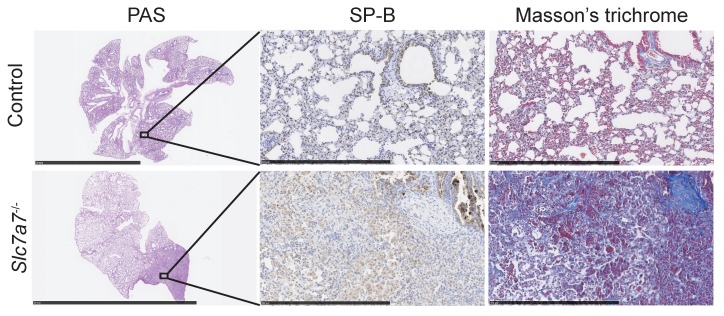
*Slc7a7^−/−^* develops pulmonary alveolar proteinosis (PAP). Sections of periodic acid–Schiff (PAS)-stained lungs reveal the classic histological features of PAP in *Slc7a7^−/−^* animals. These features were not present in control mice. Serial sections of lung were stained with SP-B antibody and Masson’s trichrome. Lipoproteinaceous material accumulated within the alveoli was PAS and surfactant protein (SP)-B positive. Pulmonary fibrosis was observed in the *Slc7a7^−/−^* mouse, as revealed by blue staining, which indicates collagen deposition. Scale bar = 5 µm for PAS images and scale bar = 500 µm for the rest.

One of the most life-threatening complications in LPI patients is pulmonar alveolar proteinosis (PAP). This condition is characterized by surfactant accumulation caused by a dysfunction of alveolar macrophages (AMs), which catabolize surfactant [38]. In PAP, foamy and enlarged AMs can be observed by bronchoalveolar lavage cytology. Lung histology revealing collapsed alveoli with surfactant accumulations is the gold standard for diagnosis in experimental models of PAP. *Slc7a7^−/−^* developed PAP in ~30% of the animals (i.e., 7 out of the 21 mice studied), a similar percentage as in LPI patients, which ranges from 10–60% in the different LPI cohorts [39,40]. Thus, PAS-positive material was found inside alveoli and was further confirmed by immunohistochemistry against surfactant protein B (SP-B, Figure 4). We also detected high levels of fibrosis (Masson’s trichrome staining) within the PAP lesions (Figure 4). Surfactant accumulations in the lungs of *Slc7a7^−/−^* animals were not clearly observed prior to day 25 post LPI induction (mild PAP signs were detected in three out of seven mice studied). Because of the low survival rate on animals without citrulline treatment, we could only analyze three animals at day 50, in which only one developed PAP. Five animals out of eleven *Slc7a7^−/−^* mice, treated with citrulline, developed PAP 50–60 days after tamoxifen induction. Given the limitations of the model, we cannot appreciate whether citrulline is involved in PAP development.

In an attempt to find differences between *Slc7a7^−/−^* mouse that develop PAP and the ones that do not show lung alterations, we compared both groups regarding their loss of body weight, which is a clear marker of the severity of LPI. No differences in the percentage of body weight loss were found between *Slc7a7^−/−^* animals with or without PAP development (Supplementary Figure S5), suggesting that PAP is not strictly related to LPI severity, and metabolic derangement does not impact PAP development.

**Figure 5:**
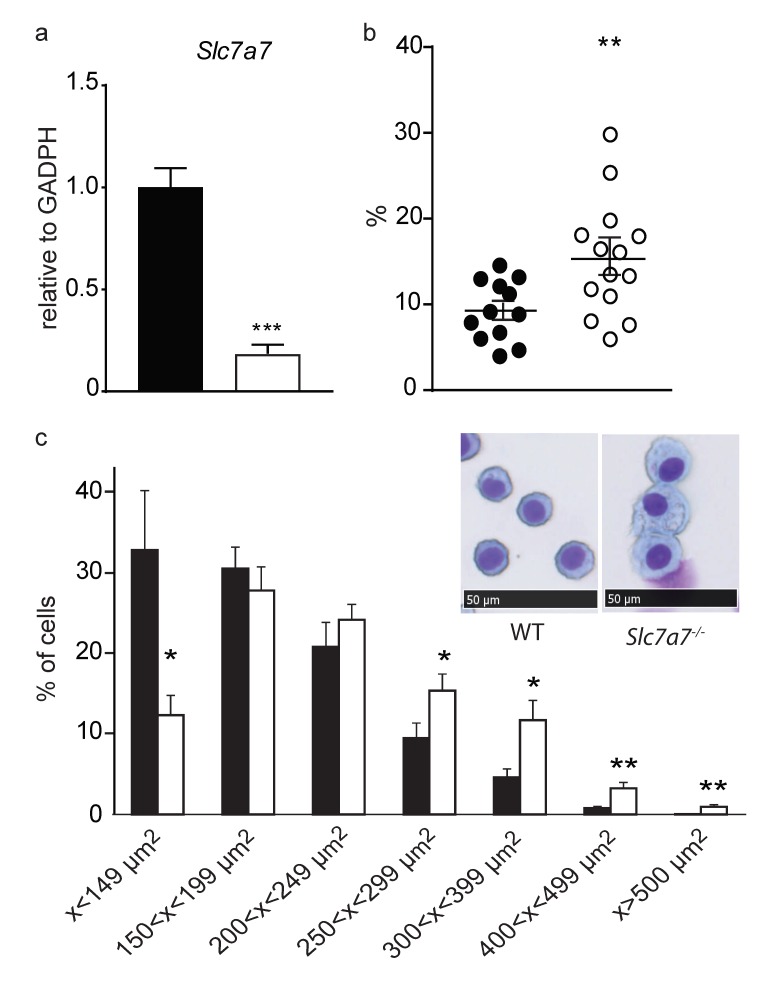
Analysis of alveolar macrophages. (**a**) y^+^LAT1 mRNA expression is strongly reduced in AMs from *Slc7a7^−/−^* mice (white bars) compared to controls ones (black bars); (**b**) Percentage of foamy AMs is increased in *Slc7a7^−/−^* animals (white circles); (**c**) AMs from *Slc7a7^−/−^* mice have a bigger area than those from controls (black bars) and are foamy as expected for surfactant accumulation (inset panels). Mice were fed with an 8% protein diet supplemented with citrulline for 15–55 days. Data corresponds to the mean ± SEM of 12 control and 14 *Slc7a7^−/−^* mice. Statistical significance * *p* < 0.05, ** *p* < 0.01, *** *p* < 0.001 was analyzed using a Student’s *t*-test. Cytological preparations of bronchoalveolar-lavage fluid were stained with panoptic. Scale bar = 50 µm.

AMs obtained by broncho-alveolar lavage (BAL) confirmed decreased expression of *Slc7a7* mRNA (to ~20%) in *Slc7a7^−/−^* mice (Figure 5a). A high percentage of foamy AMs were observed in these animals (Figure 5b). Nevertheless, only 5 out of 14 *Slc7a7^−/−^* animals showed an increased percentage of foamy AM compared to controls, which parallels the PAP penetrance found by lung histology. The increased presence of foamy AM in this mouse model was also reflected by a clear shift to a higher area range of the AM population, thereby confirming that these cells are enlarged in *Slc7a7^−/−^* animals (Figure 5c).

## 3. Discussion

Here we report the first viable animal model of LPI, the tamoxifen-induced ablation by UBC-Cre-ERT2 of *Slc7a7* in mice (*Slc7a7^−/−^*). This model reproduces the hallmarks of human LPI [14]: the renal reabsorption and the intestinal malabsorption of CAA results in hypoargininemia, which compromises the urea cycle leading to hyperammonemia and hyperglutaminemia. At odds with human LPI [14], *Slc7a7^−/−^* mice showed higher hyperexcretion of arginine and ornithine than that of lysine. Because tubular reabsorption and intestinal reabsorption of lysine is moderately and dramatically affected, respectively, in *Slc7a7^−/−^* mice, our results suggest that y^+^LAT1/CD98hc has a more prominent role in the tubular reabsorption of lysine in humans than in mice. Administration of citrulline (1 mg/mL of drinking water), a treatment used in human LPI that renders arginine and ornithine in the tubular epithelial cells of the kidney [35], rescues the mouse phenotype, thereby supporting the above indicated mechanism of pathophysiology. *Slc7a7^−/−^* mice also presented neurological alterations (astrocytosis and brain edema) with reduced food intake, body and WAT weights, and survival. Interestingly, citrulline supplementation partially or totally restored all the parameters supporting the hypothesis that hypoargininemia and the urea cycle defect underlie the neurological phenotype that compromises viability of the LPI mouse. *Slc7a7^−/−^* mice also, like human patients, develop pulmonary alveolar proteinosis (PAP) [17]. Unfortunately, the low viability of the model without citrulline treatment precludes assessing whether the metabolic derangement contributes to PAP development.

Since the first description of LPI in Finland in 1965 by Perheentupa and Visakorpi [41], more cases have been reported worldwide [16]. The clinical symptoms of the disease appear after weaning, which can be explained by endogenous arginine biosynthesis in mammals. Adult mammals synthesize arginine from citrulline mainly in the proximal tubules of the kidney [42]. The endogenous biosynthesis of arginine covers daily requirements under steady state conditions. However, supplementation of arginine from the diet may become necessary when demand increases [43]. In addition, it seems that during the weaning period of mammals, the supply of arginine from milk does not appear to meet body requirements and a high degree of endogenous synthesis is needed [44]. Endogenous arginine biosynthesis during this period occurs in the intestine rather than in the kidney. In suckling rats, it has been shown that small intestine enterocytes express arginosuccinate synthetase 1 (Ass1) and arginosuccinate liase (Asl), the enzymes required for arginine biosynthesis from citrulline. After weaning, the enterocytes lose the expression of these enzymes and start to express arginase 1 (Arg1), thereby switching their capacity for arginine synthesis to capacity for arginine degradation [45]. This physiological process may be shared with humans, as the destruction of enterocytes in necrotizing colitis in preterm neonates also results in a selective decrease in circulating arginine [46]. It could therefore be hypothesized that this metabolic switch in arginine biosynthesis after weaning is responsible for the appearance of the clinical symptoms in LPI. 

The low survival and the quick loss of body weight of *Slc7a7^−/−^* mice is possibly due to a multiorgan failure caused by the defect of the urea cycle, as can be observed in other animal models of urea cycle disorders, such as that of argininosuccinic aciduria (Asl knock out mouse model), which has a very similar phenotype. Moreover, treatment of those animals with ammonia scavengers led to an improvement in the urea cycle similar to that observed in *Slc7a7^−/−^* mice treated with citrulline [47]. Indeed, this severe phenotype can be specifically assigned to hyperammonemia, as mice lacking Arg1 develop hyperammonemia and hyperargininemia and also show a similar loss of body weight and low survival [48]. 

As in human patients, only around 30% of *Slc7a7^−/−^* animals develop PAP, suggesting the influence of other modulatory factors not yet known. Because y^+^LAT1 is expressed in macrophages [23] and metabolic derangement seems not enough to cause PAP to all the animals, some studies pointed to macrophages as playing a central role in the PAP disease. It is also known that granulocyte-macrophage colony stimulating factor (GM-CSF) is critical for AM terminal differentiation and pulmonary surfactant homeostasis [22]. Unfortunately, the use of inhaled recombinant GM-CSF as a therapy in LPI-related PAP patients has shown controversial outcomes [24]. In this line, a LPI patient relapsed after heart-lung transplantation for severe PAP-associated respiratory insufficiency and after a period of clinical remission, the patient finally died [49]. This observation allow us to hypothesize that cells from bone marrow that colonize the lung after the transplantation and/or metabolic derangement caused by LPI are at the basis of PAP pathology. Specific ablation of *Slc7a7* in macrophages would be necessary to determine the autonomous role of AMs in PAP development in LPI.

The *Slc7a7^−/−^* mouse represents the first viable experimental model of LPI, opening the way to dissect the molecular mechanisms of pathophysiology of the immune-like complications of the disease.

## 4. Materials and Methods 

### 4.1. Animal Care, Generation of Animal Model and Diet Treatments

All animal work was approved and conducted following established guidelines. This project (DARP n°9177) has been approved by the Institutional Animal Care and Use Committee of Parc Científic de Barcelona (IACUC-PCB), which considers that it complies with standard ethical regulations and meets the requirements of current applicable legislation (RD 53/2013 Council Directive; 2010/63/UE; Order 214/1997/GC). C57BL/6 mice were purchased from Envigo, Madison, WI, USA. The *Slc7a7* loxP mice generated by Genoway (Lyon, France) were bred with UBC-Cre/ERT2 (Jax 008085), [30] to excise exons 3 and 4 of Slc7a7. Homozygous y^+^LAT1 floxed mice with UBC-Cre-ERT2 were generated and were backcrossed for >12 generations with C57BL/6J by our group. After tamoxifen administration, the resulting mice were called *Slc7a7^−/−^.* Control animals were *Slc7a7* ^loxP/loxP^ with no expression of Cre protein. Control and *Slc7a7^−/−^* animals were littermates. Male mice aged 12 weeks were used. All mice were kept under stable temperature and humidity conditions, with 12 h light–dark cycles and free access to food and water. Animals were fed standard diet (Teklad global 14% protein rodent maintenance diet, Envigo, Madison, Wisconsin USA) until tamoxifen induction, which was achieved by means of a tamoxifen diet for one week (Teklad CRD TAM400/CreER, TD.55125, Envigo, Madison, Wisconsin USA) (400 mg of tamoxifen/kg of chow) in groups of 2–5 mice per cage. After the induction period, animals were kept on a low protein diet (8% casein, D06111601i Research Diets) for 7–10 days supplemented or not with 1 g/l l-citrulline (C7629, Sigma-Aldrich, St.Louis, MO, USA) in drinking water in groups of 2–5 mice per cage. Mice were anesthetized using isoflurane and sacrificed by cervical dislocation at the times indicated in the figure legends.

### 4.2. Genotyping

Mouse genotype was confirmed by multiplex-PCR using genomic DNA form the tail. The primers used were: *Slc7a7* genotyping primers 5’-AGATTCCTGATCGAGCACCTTCTTATCAC-3’ and 5’-CTTTGTATTGCTTTTCCATTCCCAGATACC-3’ amplified 741 base pair (bp) wild-type and 886 bp loxP DNA fragments. Cre (UBC) primers 5′ GACATGTTCAGGGATCGCCAGGCG-3′, 5’-GACGGAAATCCATCGCTCGACCAG-3’ amplified 597 bp DNA fragment from Cre gene. DNA polymerase was from Biotools.

### 4.3. Metabolic Studies

*Slc7a7^−/−^* mice were individually housed in metabolic cages (Techniplast; Usine d’Alimentation Rationnelle, Buguggiate, Italy) for 4 days, during which time they received low protein diet. Body weight, food and water intake, and urine output were monitored daily. Twenty-four-hour urine samples were collected in the presence of 10% thymol (Sigma-Aldrich) in isopropanol to prevent bacterial degradation of NH_4_, and kept at −20 °C until further analysis. On the last experimental day, intracardiac puncture was performed under deep anaesthesia (IsoFlo, Esteve Veterinaria, Barcelona, Spain) to obtain up to 1 mL of blood. Blood was collected in EDTA-tubes and plasma was obtained by centrifugation at 3000 rpm for 10 min at 4 °C, and stored at −80 °C until analysis. Organs of interest (i.e., epididymal white adipose tissue, liver, kidney, spleen and lung) were weighed, frozen in liquid nitrogen and stored at −80 °C until further processing for protein extraction.

### 4.4. Oral Gavage

The mice were fasted for 6 h with free access to water. Oral gavage was preformed using a specialized gavage needle (Sigma-Aldrich) affixed to a 1-mL syringe. Briefly, animals were gently restrained, and the gavage was carefully passed through the mouth, down to the depth of the last rib (~stomach). After obtaining a basal blood sample of ~30 μL from the tail vein in a EDTA-tubes, mice were given an oral dose of L-lysine (1 g/Kg of body weight, Sigma-Aldrich, L5501) or glucose (1 g/Kg of body weight, Sigma-Aldrich, G8270) in a volume of ~250 μL, and a second and third blood sample was obtained at 15 and 30 min. EDTA was used as an anticoagulant.

### 4.5. Amino Acid and Creatinine Analysis

Amino acid and creatinine concentrations in plasma and urine samples were determined as reported elsewhere [50]. Briefly, amino acids were determined by ion exchange chromatography with ninhydrin derivatization and spectrometric detection (Biochrom 30, Chromsystems, Cambridge, UK). Plasma and urine samples (300 µL) were deproteinized with sulphosalycilic acid containing L-norleucine as internal standard (final concentration 100 µmol/L). After centrifugation, 200 µL of supernatant were adjusted to pH = 2.1 with lithium hydroxide, and then, injected onto the liquid chromatograph. Creatinine concentration was determined by an automated spectrophotometric assay in the Architect c8000 analyzer (Abbott, Illinois, IL, USA). Urinary orotic acid was analyzed by a spectrometric procedure (458 nm), by reacting with para-dimethylaminobenzaldehyde. The formulas used to calculate the glomerular filtration rate; renal clearance and tubular reabsorption are described in the Supplementary Equations.

### 4.6. Brain Water Content

Mice were anesthetized using isoflurane, sacrificed by cervical dislocation and the brain was rapidly removed. Brains were weighed (w1), placed in an oven (95 °C for 48 h) and reweighed (w2). Percent brain water content was calculated (Supplementary Equation S4). 

### 4.7. Protein Analysis

Protein analysis was done by western blotting using total membrane samples. Frozen tissues were homogenized in membrane buffer (25 mM HEPES, 250 mM sacarose, 4 mM EDTA and protease inhibitors (Protease Inhibitor Cocktail Set III from Merck, Darmstadt, Germany, at a dilution of 1:1000). Samples were centrifuged at 10,000× *g*, 10 min, 4 °C, and the supernatant was subsequently centrifuged at 20,000× *g* for 1 h at 4 °C. The whole pellet was resuspended in 200 μL membrane buffer using a 25 G syringe. Samples were quantified using the Pierce BCA Protein Assay kit (Thermo Scientific, Ref: 23225, Waltham, MA, USA). Membrane proteins (50 μg) were resolved in 10% acrylamide gels for SDS-PAGE and then transferred to Immobilon membranes (Merk-Millipore, Darmstadt, Germany). Polyclonal rabbit antibody against mouse SLC7A7 protein was generated using an antigen against the N-terminal region (peptide sequence: CQHEADDGSALGDGASP) (Supplementary Figure S1c) [51]. Serum extracts from inoculated rabbits were purified with peptide column (Sulfolink coupling gel, 20401, Thermofisher, Waltham, MA, USA) following the manufacturer’s protocol. Primary antibodies were probed overnight at 4 °C at dilutions of 1:1000 (y^+^LAT1) and 1:10,000 dilution (β-actin, Sigma). Proteins were detected by the ECL reaction (GE Healthcare Ref: RPN2232, Chicago, Illinois, USA) and film exposure and quantified by scanning densitometry. 

### 4.8. Histological Sample Preparation and Analysis

Lungs were fixed in 4% paraformaldehyde for 24 h at 4 °C and embedded in paraffin blocks. The tissue was cut into 5 μm sections, air-dried and then stained with periodic acid–schiff (PAS) and Masson’s trichrome (Dako-Agilent, Santa Clara, CA, USA) following standard procedures. 

Immunohistochemistry for surfactant protein B (SP-B) was performed manually. Formalin-fixed paraffin-embedded lung sections were cut in 3 µm sections and dried at 60 °C overnight. Immunohistochemistry was performed using an Autostainer Plus (Dako-Agilent). Prior to immunohistochemistry, sections were dewaxed and antigen retrieval was done with Tris-EDTA buffer pH 9 for 20 min at 97 °C using a PT Link (Dako-Agilent). Quenching of endogenous peroxidase was performed by 10 min of incubation with peroxidase-blocking solution (Dako REAL S2023). Unspecific unions were blocked using 3% of goat normal serum (Life technology 16210064, Carlsbad, CA, USA) for 20 min. Primary antibody Rabbit Anti-mature SP-B (Seven Hills Bioreagents WRAB-48604, Cincinnati, OH, USA) at 1:1500 was incubated for 60 min at room temperature. Ready to use Bright Vision Poly-HRP-Anti Rabbit IgG Biotin-free (Immunologic, DPVR-110HRP, Duiven, Netherlands) served as secondary antibody. Antigen–antibody complexes were revealed with 3-3′-diaminobenzidine (K3468, Dako), with the same time exposure (1 min).Sections were counterstained with hematoxylin (Dako, S202084) and mounted with mounting medium, toluene-free (CS705, Dako) mounting medium using a Dako CoverStainer. Specificity of staining was confirmed by omission of the primary antibody. Images were acquired with NanoZoomer-2.0 HT C9600 digital scanner (Hamamatsu, Hamamatsu, Shizuoka, Japan) equipped with a 20× objective. All images were visualized with the NDP.view 2 U123888-01 software (Hamamatsu, Photonics, Hamamatsu, Shizuoka, Japan), and with a gamma correction set at 1.8 in the image control panel of the NDP.view 2 U12388-01 software (Hamamatsu, Photonics, Hamamatsu, Shizuoka, Japan). 

### 4.9. Panoptic Staining of Cells from Bronchoalveolar Lavage 

Bronchoalveolar lavage (BAL) was performed by flushing the lungs three times with 1 mL of saline solution. BAL cells were then obtained by centrifugation (800 rpm for 15 min at RT). Cytospin preparations were prepared and stained with panoptic staining (HS005, Cypress Diagnostics, Hulshout, Belgium). Brightfield images were acquired with Nikon E800 (Nikon, Shinagawa, Tokyo, Japan) and Olympus DP72 (Olympus, Hachioji-shi, Tokyo, Japan). Areas of AM were calculated with ImageJ (Fiji, Dresden, Germany). 

### 4.10. Gene Expression

Gene expression analysis was done by reverse transcription quantitative PCR (RT-qPCR) in combination with real time PCR using y^+^LAT1 primers: y^+^LAT1 CreUBC Fw: 5’-TCAACAGCACCAAGTATGAAGTG-3’ and y^+^LAT1 CreUBC Rw: 5’-AGCCCAGATGACCAGTGAGA-3’. For the RT reaction, 2 µg of RNA was reverse-transcribed into cDNA using the SuperScript II kit (Invitrogen, Waltham, Massachusetts, USA) following the manufacturer’s conditions. Real time qPCR was performed using Power SYBR Green PCR Master Mix (ThermoFisher) and analyzed in a QuantStudio 6 Flex Real-Time PCR System (ThermoFisher Scientific). β-actin was used as internal control. Quantification cycle (C_q_) values were determined and 2^−ΔCq^ values were calculated. 

## 5. Conclusions

In conclusion, we present here the first viable animal model of Lysinuric Protein Intolerance (*Slc7a7^−/−^*) that recapitulates the main hallmarks of the human disease. This includes the hyperexcrecion and malabsorption of cationic aminoacids (CAA), which leads to a decrease in plasma CAA and defective urea cycle. Moreover, hyperammonemia and neurological impairment are also developed in *Slc7a7^−/−^* model. As in LPI patients, citrulline treatment improves the metabolic derangement and survival. Strikingly, LPI mouse model reproduces pulmonary alveolar proteinosis, one of the most life-threatening complication of the pathology. Taking advantage of the generated mouse model of human LPI, one key question for follow-up studies would be to deepen into the immune-like complications of the disease.

## Figures and Tables

**Table 1:** Percentage of tubular reabsorption of cationic amino acids.

Amino Acid	Control	*Slc7a7^-/-^*	Control + Cit	*Slc7a7^-/-^ + Cit*
ARG	99.5 ± 0.6	53.2 ± 13.4 **	97.6 ± 0.7 #	−213.3 ± 112.5 *#
ORN	99.1 ± 0.1	50.6 ± 11.7 **	99.1 ± 0.2	−51.5 ± 38.1 **#
LYS	99.7 ± 0.1	98.4 ± 0.4 *	99.6 ± 0.1	94.5 ± 1.5 **#

The percentage of tubular reabsorption was estimated using Supplementary Equation S3. Data correspond to the mean ± SEM of 6 animals per group. Statistical significance * *p* < 0.05, ** p<0.01 vs. control and # *p* < 0.05 vs. citrulline treatment (+ Cit) was analyzed using a Student’s *t*-test. L-amino acids are indicated in the three-letter code.

## Supplementary Materials

Supplementary materials can be found at https://www.mdpi.com/1422-0067/20/21/5294/s1.

## Abbreviations

AM	Alveolar macrophages
CAA	Cationic amino acids
eWAT	Epydidimal white adipose tissue
GFAP	Glial fibrillary acidic protein
GFR	Glomerular filtration rate
GM-CSF	Granulocyte-macrophage-colony stimulating factor
HHH	Hyperornithinemia hyperammonemia homocitrullinuria
LPI	Lysinuric protein intolerance
PAP	Pulmonary alveolar proteinosis
PAS	Periodic acid–Schiff staining
TR	Tubular reabsorption
