# Supplementary material for: Inducible Slc7a7 Knockout Mouse Model Recapitulates Lysinuric Protein Intolerance Disease
Source: Int J Mol Sci. 2019 Oct 24;20(21):5294. doi: 10.3390/ijms20215294 (PMC6862226; doi:10.3390/ijms20215294)
Supplement: Supplementary file 1 [file ijms-20-05294-s001.pdf]

Supplementary Materials:

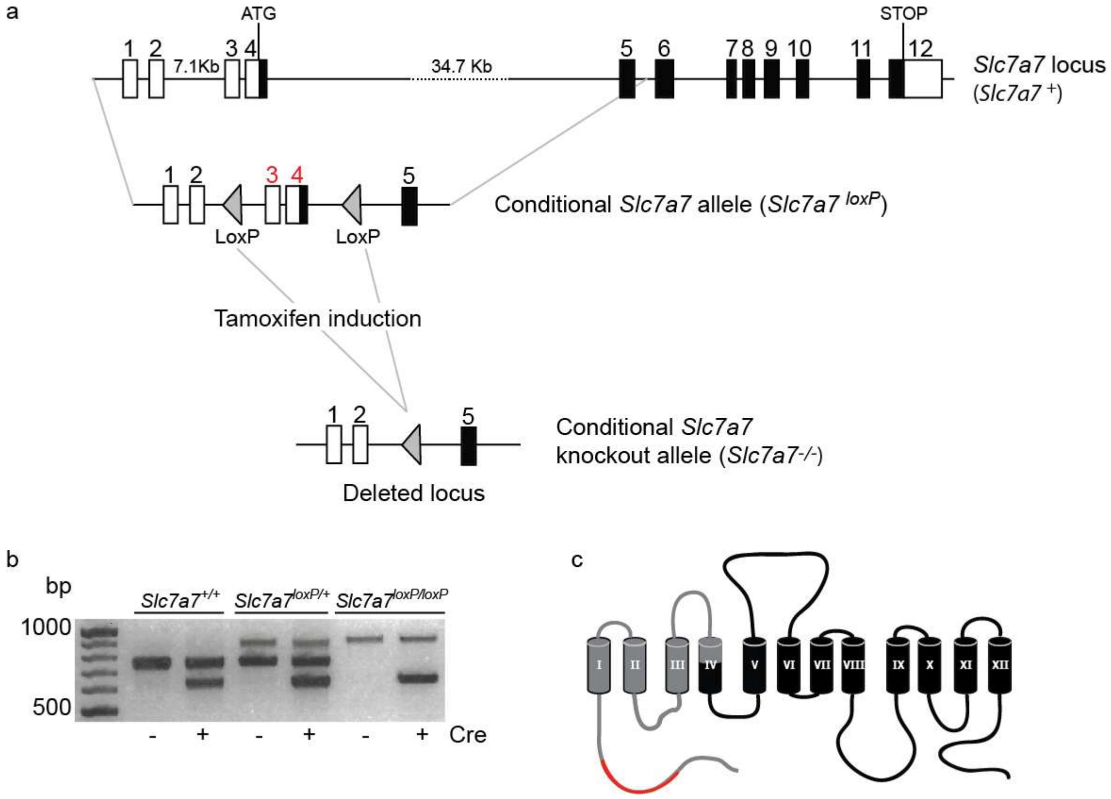

Supplementary Figure S1: Conditional *Slc7a7* gene deletion and effect on protein. (**a**) Schematic representation of *Slc7a7* conditional strategy. First row represents wild-type murine Slc7a7 with open boxes for untranslated exons and black boxes for codifying exons. Exons 3 and 4 are flanked by loxP sequences (triangles) generating a conditional *Slc7a7* loxP, in which, after tamoxifen induction, an *in vivo* Cre-mediated excision occurs, leading to a non-functional protein (*Slc7a7*$^{-/-}$); (**b**) Genotyping of control (*Slc7a7*$^{+/+}$), *Slc7a7*$^{loxP/+}$ and *Slc7a7*$^{loxP/loxP}$ mice with or without Cre by multiplex PCR amplification of genomic DNA. Expected bands of 741 bp and 886bp are detected for the *Slc7a7*$^{+}$ and *Slc7a7*^{loxP} alleles. Amplification of the Cre band is detected at 597 bp; (**c**) Schematic topology of y^{+}LAT1 protein. Deleted region in the *Slc7a7*$^{-/-}$ mouse is depicted in gray and the epitope used to generate rabbit anti-mouse SLC7A7 antibody is showed in red.

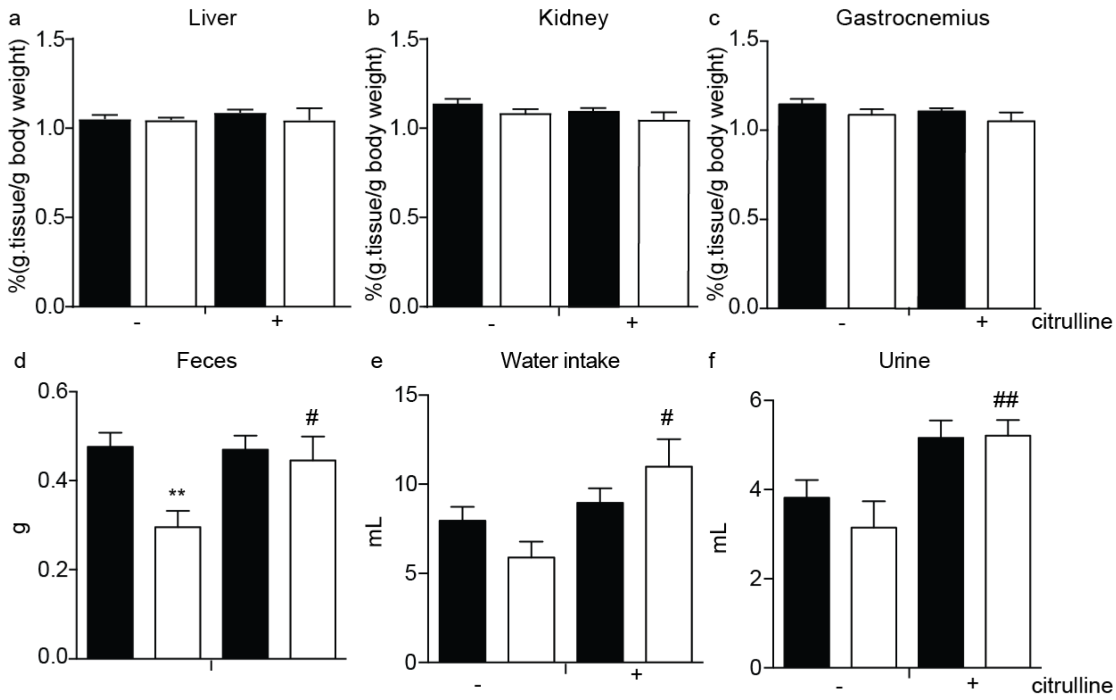

Supplementary Figure S2. **Metabolic parameters and organ weights.** (**a-c**) Liver, kidney and gastrocnemius weight of control (black bars) and *Slc7a7*$^{-/-}$ (white bars) animals fed with an 8% of protein diet with or without citrulline supplementation; (**d**) amount of feces excreted in 24h; (**e**) water intake, in mL in 24h; (**f**) volume of urine excreted in 24h.

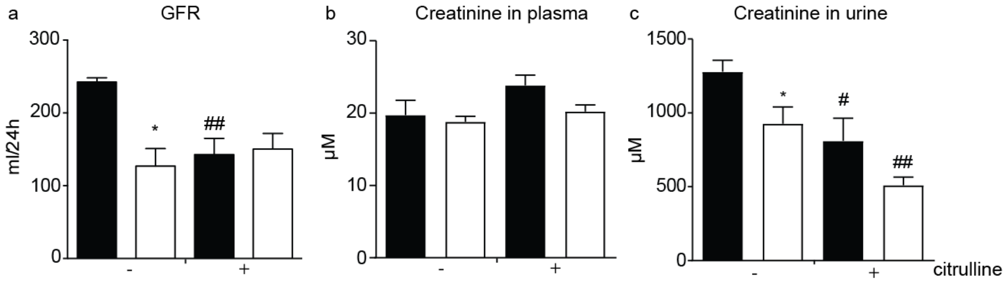

Supplementary Figure S3. Glomerular filtration rate. (**a**) Glomerular filtration rate was estimated using Equation 2 (see Supplementary equation 2) in control (black bars) and *Slc7a7*$^{-/-}$ (white bars) mice; (**b-c**) Creatinine concentration (μM) in plasma and urine are also shown. In all cases, mice were analyzed after 7-10 days on an 8% protein diets with or without citrulline in drinking water. Data corresponds to the mean±SEM of 6 mice per group. Statistical significance *p < 0.05 vs. control. #p<0.05, ##p<0.01 vs. citrulline treatment was analyzed using a Student's t-test.

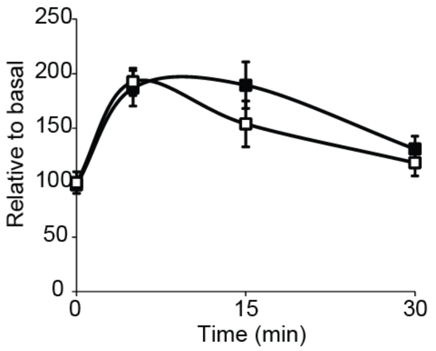

Supplementary Figure S4. Oral gavage of 1g glucose/kg was performed in control (black squares) and *Slc7a7*$^{-/-}$ (white squares) animals. Glucose was measured before the oral gavage and at 5, 15 and 30 minutes after the glucose administration. Data corresponds to the mean±SEM of 6 mice per group.

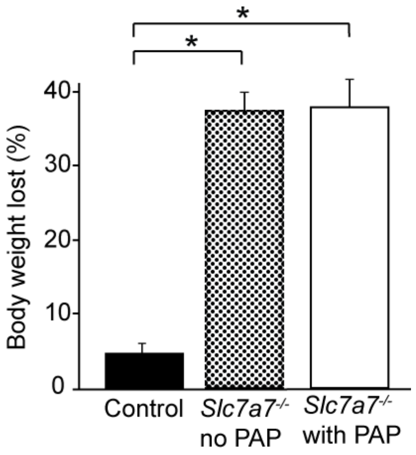

Supplementary Figure S5. PAP development. Analysis of PAP development compared to severity of LPI measured as body weight lost at the day of sacrifice. Data corresponds to the mean±SEM of 15 control (n=7 treated with citrulline and n=8 without citrulline treatment) and 20 *Slc7a7*$^{-/-}$ (n=10 treated with citrulline and n=10 not treated) mice. Statistical significance *$p < 0.05$ vs. control was analyzed using a Student's t-test. Animals were on a low protein diet with or without citrulline supplementation for 15-55 days.

Supplementary Table S1. Amino acid concentration in plasma. Plasma concentrations (μM) were determined in mice with the indicated genotypes at 12 months of age and after 10 days on an 8% protein diet. Data corresponds to the mean±SEM of 6 mice per group. Statistical significance *$p < 0.05$, **$p<0.01$, ***$p<0.001$, ****$p<0.0001$ vs. control. #$p<0.05$, ##$p<0.01$, ###p<0,001 vs. citrulline supplementation was analyzed using a Student's t-test. Amino acids are designated with the three-letter code.

Amino acid	Control	*Slc7a7*$^{-/-}$	Control + Cit	*Slc7a7*$^{-/-}$ + Cit
THR	119.3 ± 14.5	181.9 ± 1.5 **	254.2 ± 19.5 ###	189.7 ± 27.2
SER	84.5 ± 6.2	134.3 ± 12.5 **	186.6 ± 13.5	144.6 ± 16.5
ASN	34.5 ± 2.3	56.0 ± 7.4 *	50.9 ± 5.2 #	61.4 ± 5.2
GLN	470.5 ± 44.4	1005 ± 90.1 ***	591.4 ± 93.9 ###	839.5 ± 112.7
GLY	144.4 ± 19.9	244.9 ± 35.3 *	268.6 ± 25.6 ##	210.7 ± 11.9
ALA	279.2 ± 23.7	528.5 ± 76.3 **	747.8 ± 98.9 ###	451.4 ± 53.8 *
CTR	44.4 ± 4.4	84.1 ± 11.2 **	86.1 ± 6.8 ###	119.2 ± 11.1 * #
VAL	136.4 ± 18.3	158.4 ± 15.1	190.8 ± 11.0 #	135.5 ± 11.0 **
MET	37.4 ± 3.4	62.0 ± 6.2 **	66.1 ± 8.5 #	61.1 ± 9.2
ILE	53.2 ± 7.0	56.8 ± 6.1	84.2 ± 4.1 ##	53.5 ± 2.3 ****
LEU	97.3 ± 17.0	113.3 ± 11.7	132.5 ± 7.5	96.1 ± 6.5 **
HIS	50.8 ± 6.7	90.0 ± 10.8 *	85.7 ± 9.0 #	62.0 ± 4.9 * #
TYR	38.8 ± 2.4	58.7 ± 5.8 *	53.9 ± 1.9 ##	71.2 ± 8.3
PHE	49.0 ± 6.1	75.9 ± 7.1 *	79.6 ± 8.5 #	58.2 ± 2.9 * #
PRO	57.6 ± 2.7	96.7 ± 7.6 ***	129.0 ± 27.5 #	92.1 ± 8.2
ASP	17.1 ± 1.2	17.1 ± 3.5	34.6 ± 1.4	19.6 ± 4.8
GLU	52.1 ± 7.9	33.1 ± 3.3	65.4 ± 20.6	56.80 ± 11.1
ARG	47.4 ± 6.8	11.4 ± 3.6 ***	72.0 ± 17.1	31.1 ± 5.3 * #
ORN	32.4 ± 1.6	33.7 ± 7.0	104.5 ± 16.3 ##	31.2 ± 3.4 **
LYS	464.6 ± 82.2	189.3 ± 20.1 **	321.9 ± 8.4	159.2 ± 15.6 ****

Supplementary Table S2. Excretion of amino acids in urine. Excretion, expressed as nmols of the indicated amino acid per gram of body weight in a 24-hour sample, was assessed in mice with the indicated genotypes at 12 months of age and after 7-10 days on an 80% protein with or without citrulline (Cit) supplementation. Data corresponds to the mean±SEM of 6 mice per group. Statistical significance *p < 0.05, **p<0.01, ***p<0.001 vs. control. #p<0.05, ##p<0.01, ###p<0,001 vs. citrulline supplementation was analyzed using a Student's t-test. Amino acids are designated with the three-letter code.

Amino acid	Control	*Slc7a7*$^{-/-}$	Control + Cit	*Slc7a7*$^{-/-}$ + Cit
THR	18.9 ± 4.1	25.1 ± 4.9	30.6 ± 4.7	47.2 ± 5.8 #
SER	4.3 ± 0.4	5.6 ± 1.0	4.9 ± 0.8	8.7 ± 1.3 *
ASN	4.4 ± 0.6	5.8 ± 1.1	5.8 ± 1.1	8.0 ± 1.1
GLN	15.8 ± 1.3	116.2 ± 30.0 **	24.8 ± 8.8	328.9 ± 69.8 ** #
GLY	15.0 ± 2.1	10.3 ± 1.9	17.2 ± 1.3	12.7 ± 1.2 *
ALA	9.6 ± 1.5	9.8 ± 1.9	10.4 ± 1.0	16.5 ± 2.8
CTR	8.5 ± 1.6	13.0 ± 5.7	8.6 ± 2.0	92.2 ± 20.0 **##
VAL	4.2 ± 0.6	4.3 ± 1.4	4.1 ± 0.7	5.7 ± 0.9
MET	9.9 ± 1.7	22.8 ± 4.6 *	15.7 ± 4.3	46.9 ± 7.9 **#
ILE	1.3 ± 0.2	1.3 ± 0.7	2.1 ± 0.4	2.7 ± 0.4
LEU	2.8 ± 0.2	2.5 ± 1.1	6.8 ± 2.4	4.9 ± 1.0
HIS	1.9 ± 0.2	4.9 ± 1.4	3.0 ± 0.5	14.4 ± 18.4 ** #
TYR	3.4 ± 0.5	3.6 ± 1.0	5.1 ± 0.5	10.3 ± 1.6 **##
PHE	2.7 ± 0.3	2.0 ± 0.6	3.2 ± 0.5	3.7 ± 0.4 #
PRO	6.7 ± 2.0	21.1 ± 8.0	17.9 ± 7.6	40.0 ± 2.7
ASP	2.6 ± 0.6	2.5 ± 1.9	2.5 ± 0.4	4.0 ± 1.8
GLU	2.5 ± 0.4	4.2 ± 1.0	3.9 ± 0.9	9.4 ± 1.2 **##
ARG	1.8 ± 0.3	72.3 ± 30.9 *	8.2 ± 3.4	667.9 ± 159.0 **##
ORN	2.7 ± 0.5	64.7 ± 26.4 *	4.3 ± 1.1	260.6 ± 61.0 **#
LYS	9.3 ± 0.6	9.5 ± 0.6	7.5 ± 0.8	46.6 ± 8.0 ***###

Supplementary Table S3: Renal clearance of amino acids. Renal clearance (mL/24h·g body weight) was calculated in control and *Slc7a7*$^{-/-}$ mice at 12 months of age and after 7-10 days on an 8% protein diet with or without citrulline (Cit) supplementation. Data corresponds to the mean±SEM of 6 mice per group. Statistical significance *p < 0.05, **p<0.01, ***p<0.001 vs. control. #p<0.05, ##p<0.01 vs. citrulline supplementation was analyzed using a Student's t-test. Amino acids are designated with the three-letter code.

Amino acid	Control	*Slc7a7*$^{-/-}$	Control + Cit	*Slc7a7*$^{-/-}$ + Cit
THR	0.19 ± 0.05	0.14 ± 0.03	0.14 ± 0.01	0.28 ± 0.04 *#
SER	0.05 ± 0.01	0.04 ± 0.01	0.04 ± 0.00	0.06 ± 0.01 *
ASN	0.16 ± 0.04	0.12 ± 0.03	0.12 ± 0.01	0.17 ± 0.03
GLN	0.03 ± 0.00	0.11 ± 0.03 *	0.03 ± 0.00	0.42 ± 0.09 **##
GLY	0.11 ± 0.01	0.05 ± 0.01 **	0.10 ± 0.02	0.06 ± 0.01
ALA	0.03 ± 0.00	0.02 ± 0.00	0.02 ± 0.00 ##	0.03 ± 0.00 *
CTR	0.19 ± 0.02	0.10 ± 0.03 *	0.11 ± 0.02 #	0.76 ± 0.14 **##
VAL	0.02 ± 0.00	0.03 ± 0.01	0.03 ± 0.00 #	0.04 ± 0.00
MET	0.29 ± 0.03	0.38 ± 0.09	0.33 ± 0.07	0.83 ± 0.15 *#
ILE	0.03 ± 0.01	0.03 ± 0.01	0.04 ± 0.01	0.04 ± 0.01
LEU	0.03 ± 0.00	0.03 ± 0.01	0.10 ± 0.04	0.05 ± 0.00
HIS	0.03 ± 0.00	0.06 ± 0.01	0.05 ± 0.00	0.23 ± 0.05 **##
TYR	0.07 ± 0.01	0.06 ± 0.02	0.09 ± 0.01	0.13 ± 0.03
PHE	0.05 ± 0.00	0.03 ± 0.01	0.06 ± 0.01	0.06 ± 0.01 #
PRO	0.11 ± 0.02	0.24 ± 0.10	0.23 ± 0.03	0.44 ± 0.05 **
ASP	0.25 ± 0.08	0.26 ± 0.10	0.38 ± 0.21	0.31 ± 0.15
GLU	0.09 ± 0.00	0.08 ± 0.02	0.10 ± 0.01	0.16 ± 0.03
ARG	0.04 ± 0.00	4.38 ± 7.32 **	0.19 ± 0.05 #	27.23 ± 8.74 *#
ORN	0.07 ± 0.01	3.20 ± 1.41 *	0.07 ± 0.02	9.42 ± 2.91 **
LYS	0.03 ± 0.00	0.07 ± 0.02 *	0.03 ± 0.00	0.30 ± 0.05 ***##

Supplementary Table S4: Tubular reabsorption of neutral and acidic amino acids. The percentage of tubular reabsorption was estimated in control and *Slc7a7*$^{-/-}$ at 12 months of age and after 7-10 days on an 8% protein-content diet with (Cit) or without citrulline supplementation. Data corresponds to the mean±SEM of 6 mice per group. Statistical significance *p < 0.05, **p<0.01, ***p<0.001 vs. control. #p<0.05, ##p<0.01 vs. citrulline supplementation was analyzed using a Student's t-test. Amino acids are designated with the three-letter code.

Amino acid	Control	*Slc7a7*$^{-/-}$	Control + Cit	*Slc7a7*$^{-/-}$ + Cit
THR	98.3 ± 0.5	98.0 ± 0.2	98.1 ± 0.2	96.3 ± 0.7 *#
SER	99.4 ± 0.1	99.4 ± 0.1	99.6 ± 0.1	99.0 ± 0.2 **
ASN	98.7 ± 0.2	98.5 ± 0.2	98.0 ± 0.5	98.2 ± 0.3
GLN	99.7 ± 0.1	97.8 ± 0.3 ****	99.5 ± 0.1	94.4 ± 1.3 **#
GLY	98.8 ± 0.1	99.4 ± 0.1 *	98.8 ± 0.2	99.0 ± 0.1
ALA	99.6 ± 0.1	99.6 ± 0.1	99.7 ± 0.1	99.4 ± 0.1 **
CTR	98.1 ± 0.3	97.1 ± 0.3	98.5 ± 0.3	86.3 ± 4.0 *#
VAL	99.6 ± 0.1	99.4 ± 0.2	99.6 ± 0.2	99.3 ± 0.1
MET	96.8 ± 0.4	93.8 ± 1.8 **	96.0 ± 0.9	89.3 ± 1.4 **#
ILE	99.7 ± 0.1	99.1 ± 0.2	99.6 ± 0.1	99.7 ± 0.1 #
LEU	99.7 ± 0.1	99.8 ± 1.0	98.9 ± 0.5	99.1 ± 0.2 #
HIS	99.6 ± 0.1	98.7 ± 0.2 *	99.4 ± 0.1	96.1 ± 1.0 **#
TYR	99.2 ± 0.2	99.3 ± 0.1	98.8 ± 0.2	97.4 ± 0.7 #
PHE	99.4 ± 0.1	99.7 ± 0.1	99.3 ± 0.1	98.9 ± 0.2 ##
PRO	98.7 ± 0.2	94.6 ± 2.2	97.1 ± 0.50	92.1 ± 1.3 **
ASP	97.3 ± 0.8	96.9 ± 1.0	99.2 ± 0.1 #	96.6 ± 1.0
GLU	99.1 ± 0.2	98.4 ± 0.3	98.8 ± 0.2	97.6 ± 0.6
